# Supplementary material for: Relationships between crayfish population genetic diversity, species richness, and abundance within impounded and unimpounded streams in Alabama, USA
Source: PeerJ. 2024 Sep 24;12:e18006. doi: 10.7717/peerj.18006 (PMC11430169; doi:10.7717/peerj.18006)
Supplement: Supplemental Information 1 — Site coordinates (latitudes and longitudes in decimal degrees; map datum WGS 84), dam heights (Height; m), impoundment areas/lengths (Area [ha]; length [km]), and impoundment full and low pool elevations (m above sea level), year dam construction completed are listed. Site labels correspond to those in Fig. 1. U = unimpounded; I = impounded. Up = upstream. Dn = downstream either from an impoundment, or the midpoint in the unimpounded stream. Fval = Faxonius validus. Feri = Faxonius erichsonianus [file peerj-12-18006-s001.docx]

**Supplemental Data S1.** Site locations and distance (km) from midpoint (i.e., dam in impounded streams) for collections of *Faxonius validus* and *F. erichsonianus* in Bear Creek and Cahaba River drainages. Site coordinates (latitudes and longitudes in decimal degrees; map datum WGS 84), dam heights (Height; m), impoundment areas/lengths (Area [ha]; length [km]), and impoundment full and low pool elevations (m above sea level), year dam construction completed are listed. Site labels correspond to those in Fig. 1. U = unimpounded; I = impounded; Up = upstream; Dn = downstream either from an impoundment, or the midpoint in the unimpounded stream; Fval = *Faxonius validus*; Feri = *Faxonius erichsonianus*

| Drainage | Stream | Stream type | Site label | Stream Section | County | Latitude | Longitude | Distance | Height | Area/  length | Full/low pool | Year Completed | Focal species collected |
| --- | --- | --- | --- | --- | --- | --- | --- | --- | --- | --- | --- | --- | --- |
| Bear Creek | Rock | U | R1 | Up | Colbert | 34.60473 | -87.90642 | 13.56 |  |  |  |  | Fval |
|  |  |  | R2 | Up | Colbert | 34.59693 | -87.92230 | 11.50 |  |  |  |  | Fval/Feri |
|  |  |  | R3 | Up | Colbert | 34.60080 | -87.97946 | 4.02 |  |  |  |  | Fval/Feri |
|  |  |  | R4 | Dn | Colbert | 34.59995 | -88.03600 | 1.87 |  |  |  |  | Fval/Feri |
|  |  |  | R5 | Dn | Colbert | 34.62367 | -88.07604 | 10.49 |  |  |  |  | Fval/Feri |
|  |  |  | R6 | Dn | Colbert | 34.63284 | -88.09255 | 12.63 |  |  |  |  | Fval/Feri |
|  | Cedar | I | C1 | Up | Franklin | 34.44125 | -87.71965 | 26.71 | 29.3 | 1700/14.9 | 177/173 | 1979 | Fval/Feri |
|  |  |  | C2 | Up | Franklin | 34.46392 | -87.75135 | 19.29 |  |  |  |  | Fval/Feri |
|  |  |  | C3 | Up | Franklin | 34.47114 | -87.80230 | 11.12 |  |  |  |  | Fval/Feri |
|  |  |  | C4 | Up | Franklin | 34.48408 | -87.82598 | 5.34 |  |  |  |  | Fval/Feri |
|  |  |  | C5 | Dn | Franklin | 34.54716 | -87.97808 | 0.30 |  |  |  |  | Fval/Feri |
|  |  |  | C6 | Dn | Franklin | 34.55284 | -87.98467 | 1.69 |  |  |  |  | Fval/Feri |
|  |  |  | C7 | Dn | Franklin | 34.55990 | -87.99809 | 2.79 |  |  |  |  | Fval/Feri |
|  |  |  | C8 | Dn | Franklin | 34.54814 | -88.01796 | 6.63 |  |  |  |  | Fval/Feri |
|  | Little Bear | I | LB1 | Up | Franklin | 34.36400 | -87.73300 | 24.64 | 25.6 | 631/  7.6 | 189/185 | 1975 | Fval |
|  |  |  | LB2 | Up | Franklin | 34.37757 | -87.77609 | 20.07 |  |  |  |  | Fval/Feri |
|  |  |  | LB3 | Up | Franklin | 34.38086 | -87.80641 | 15.81 |  |  |  |  | Fval/Feri |
|  |  |  | LB4 | Up | Franklin | 34.38190 | -87.83505 | 11.60 |  |  |  |  | Fval/Feri |
|  |  |  | LB5 | Up | Franklin | 34.40267 | -87.87422 | 6.22 |  |  |  |  | Fval/Feri |
|  |  |  | LB6 | Dn | Franklin | 34.45491 | -87.98467 | 0.10 |  |  |  |  | Fval |
|  |  |  | LB7 | Dn | Franklin | 34.45600 | -87.98300 | 1.25 |  |  |  |  | Fval/Feri |
|  |  |  | LB8 | Dn | Franklin | 34.46090 | -88.00422 | 4.68 |  |  |  |  | Fval/Feri |
|  |  |  | LB9 | Dn | Franklin | 34.48800 | -88.03500 | 11.84 |  |  |  |  | Fval/Feri |
|  |  |  | LB10 | Dn | Franklin | 34.50118 | -88.04926 | 14.91 |  |  |  |  | Fval/Feri |
| Cahaba River | Shades | U | S1 | Up | Jefferson | 33.35442 | -86.93914 | 6.53 |  |  |  |  | Feri |
|  |  |  | S2 | Up | Jefferson | 33.32637 | -86.94901 | 2.69 |  |  |  |  | Feri |
|  |  |  | S3 | Dn | Jefferson | 33.30746 | -86.96230 | 2.91 |  |  |  |  | Feri |
|  |  |  | S4 | Dn | Jefferson | 33.29454 | -86.98317 | 6.39 |  |  |  |  | Feri |
|  | Little Cahaba | I | LC1 | Up | Jefferson | 33.51813 | -86.58300 | 6.80 | 16.8 | 425/  3.2 | 168/-- | 1911 | Feri |
|  |  |  | LC2 | Up | Jefferson | 33.49999 | -86.61325 | 1.28 |  |  |  |  | Feri |
|  |  |  | LC3 | Dn | Jefferson | 33.43950 | -86.69749 | 5.60 |  |  |  |  | Feri |
|  |  |  | LC4 | Dn | Jefferson | 33.43732 | -86.70172 | 6.80 |  |  |  |  | Feri |
