# Supplementary material for: Relationships between crayfish population genetic diversity, species richness, and abundance within impounded and unimpounded streams in Alabama, USA
Source: PeerJ. 2024 Sep 24;12:e18006. doi: 10.7717/peerj.18006 (PMC11430169; doi:10.7717/peerj.18006)
Supplement: Supplemental Information 2 — The total number of different haplotypes detected for each species is represented in parentheses. [file peerj-12-18006-s002.docx]

**Supplemental Data S2.** List of ISSR primers tested and used for the two focal crayfish species. Numbers listed under ‘ISSR primer name’ correspond to the UBC Primer Set 9 names (paper available on GitHub [www.github.com/btsinn/ISSRseq](http://www.github.com/btsinn/ISSRseq)). N/A = primers that did not yield polymorphic loci or reproducible gel phenotypes.

| ISSR primer name | Primer sequence (5’ to 3’) | Utility in this study | Number of loci scored |
| --- | --- | --- | --- |
| 844 | [CT]_8_RC | *F. erichsonianus* | 19 |
| 845 | [CT]_8_RG | N/A |  |
| RCG3 | [CA]_8_KC | N/A |  |
| RCG4 | [CA]_8_KG | N/A |  |
| RCG5 | [AAG]_5_YC | *F. validus* | 13 |
| RCG6 | [AAG]_5_YG | N/A |  |
| RCG7 | [CCT]_5_RC | N/A |  |
| RCG8 | [CCT]_5_RG | *F. validus* | 11 |
| RCG9 | [CAA]_5_SC | *F. erichsonianus* | 16 |
| RCG10 | [CAA]_5_SG | N/A |  |
