# Supplementary material for: Relationships between crayfish population genetic diversity, species richness, and abundance within impounded and unimpounded streams in Alabama, USA
Source: PeerJ. 2024 Sep 24;12:e18006. doi: 10.7717/peerj.18006 (PMC11430169; doi:10.7717/peerj.18006)
Supplement: Supplemental Information 3 — Numbers listed under ‘ISSR primer name’ correspond to the UBC Primer Set 9 names (paper available on GitHub www.github.com/btsinn/ISSRseq). N/A = primers that did not yield polymorphic loci or reproducible gel phenotypes. [file peerj-12-18006-s003.docx]

**Supplemental Data S3.** The National Center for Biotechnology Information’s nucleotide database accession numbers for each mtCOI haplotype, the haplotype code (Species_haplotype number) and the number of individuals from each stream with each haplotype. The total number of different haplotypes detected for each species is represented in parentheses. FV= *Faxonius validus*; FE = *Faxonius erichsonianus*.

|  | Haplotype | Bear Creek Drainage | | | Cahaba River Drainage | |  |
| --- | --- | --- | --- | --- | --- | --- | --- |
| Accession Number | code | Little Bear | Cedar | Rock | Little Cahaba | Shades | Total |
| *Faxonius validus* (28) |  |  |  |  |  |  |  |
| MN053979 | FV_Hap1 | 8 | 14 | 0 | 0 | 0 | 22 |
| MN053980 | FV_Hap2 | 31 | 7 | 0 | 0 | 0 | 38 |
| MN053981 | FV_Hap3 | 0 | 1 | 0 | 0 | 0 | 1 |
| MN053982 | FV_Hap4 | 0 | 2 | 0 | 0 | 0 | 2 |
| MN053983 | FV_Hap5 | 0 | 2 | 0 | 0 | 0 | 2 |
| MN053984 | FV_Hap6 | 12 | 11 | 0 | 0 | 0 | 23 |
| MN053985 | FV_Hap7 | 0 | 3 | 0 | 0 | 0 | 3 |
| MN053986 | FV_Hap8 | 1 | 4 | 0 | 0 | 0 | 5 |
| MN053987 | FV_Hap9 | 0 | 1 | 0 | 0 | 0 | 1 |
| MN053988 | FV_Hap10 | 0 | 1 | 0 | 0 | 0 | 1 |
| MN053989 | FV_Hap11 | 0 | 1 | 0 | 0 | 0 | 1 |
| MN053990 | FV_Hap12 | 0 | 1 | 0 | 0 | 0 | 1 |
| MN053991 | FV_Hap13 | 0 | 1 | 0 | 0 | 0 | 1 |
| MN053992 | FV_Hap14 | 0 | 1 | 0 | 0 | 0 | 1 |
| MN053993 | FV_Hap15 | 0 | 1 | 0 | 0 | 0 | 1 |
| MN053994 | FV_Hap16 | 1 | 1 | 0 | 0 | 0 | 2 |
| MN053995 | FV_Hap17 | 1 | 0 | 0 | 0 | 0 | 1 |
| MN053996 | FV_Hap18 | 1 | 0 | 0 | 0 | 0 | 1 |
| MN053997 | FV_Hap19 | 1 | 0 | 0 | 0 | 0 | 1 |
| MN053998 | FV_Hap20 | 1 | 0 | 0 | 0 | 0 | 1 |
| MN053999 | FV_Hap21 | 1 | 0 | 0 | 0 | 0 | 1 |
| MN054000 | FV_Hap22 | 0 | 0 | 27 | 0 | 0 | 27 |
| MN054001 | FV_Hap23 | 0 | 0 | 1 | 0 | 0 | 1 |
| MN054002 | FV_Hap24 | 0 | 0 | 1 | 0 | 0 | 1 |
| MN054003 | FV_Hap25 | 0 | 0 | 1 | 0 | 0 | 1 |
| MN054004 | FV_Hap26 | 0 | 0 | 1 | 0 | 0 | 1 |
| MN054005 | FV_Hap27 | 0 | 0 | 1 | 0 | 0 | 1 |
| MN054006 | FV_Hap28 | 0 | 0 | 1 | 0 | 0 | 1 |
| *Faxonius erichsonianus* (42) |  |  |  |  |  |  |  |
| MN054007 | FE_Hap1 | 0 | 17 | 0 | 0 | 0 | 17 |
| MN054008 | FE_Hap2 | 0 | 1 | 0 | 0 | 0 | 1 |
| MN054009 | FE_Hap3 | 0 | 2 | 0 | 0 | 0 | 2 |
| MN054010 | FE_Hap4 | 0 | 11 | 0 | 0 | 0 | 11 |
| MN054011 | FE_Hap5 | 0 | 3 | 0 | 0 | 0 | 3 |
| MN054012 | FE_Hap6 | 0 | 1 | 0 | 0 | 0 | 1 |
| MN054013 | FE_Hap7 | 0 | 2 | 0 | 0 | 0 | 2 |
| MN054014 | FE_Hap8 | 0 | 1 | 0 | 0 | 0 | 1 |
| MN054015 | FE_Hap9 | 0 | 1 | 0 | 0 | 0 | 1 |
| MN054016 | FE_Hap10 | 0 | 1 | 0 | 0 | 0 | 1 |
| MN054017 | FE_Hap11 | 0 | 1 | 0 | 0 | 0 | 1 |
| MN054018 | FE_Hap12 | 0 | 1 | 0 | 0 | 0 | 1 |
| MN054019 | FE_Hap13 | 0 | 1 | 0 | 0 | 0 | 1 |
| MN054020 | FE_Hap14 | 0 | 1 | 0 | 0 | 0 | 1 |
| MN054021 | FE_Hap15 | 11 | 0 | 0 | 0 | 0 | 11 |
| MN054022 | FE_Hap16 | 30 | 0 | 0 | 0 | 1 | 31 |
| MN054023 | FE_Hap17 | 1 | 0 | 0 | 0 | 0 | 1 |
| MN054024 | FE_Hap18 | 1 | 0 | 0 | 0 | 0 | 1 |
| MN054025 | FE_Hap19 | 1 | 0 | 0 | 0 | 0 | 1 |
| MN054026 | FE_Hap20 | 0 | 0 | 0 | 3 | 0 | 3 |
| MN054027 | FE_Hap21 | 0 | 0 | 0 | 4 | 0 | 4 |
| MN054028 | FE_Hap22 | 0 | 0 | 0 | 1 | 0 | 1 |
| MN054029 | FE_Hap23 | 0 | 0 | 0 | 2 | 0 | 2 |
| MN054030 | FE_Hap24 | 0 | 0 | 0 | 1 | 0 | 1 |
| MN054031 | FE_Hap25 | 0 | 0 | 0 | 2 | 0 | 2 |
| MN054032 | FE_Hap26 | 0 | 0 | 0 | 13 | 13 | 25 |
| MN054033 | FE_Hap27 | 0 | 0 | 0 | 2 | 1 | 3 |
| MN054034 | FE_Hap28 | 0 | 0 | 0 | 2 | 0 | 2 |
| MN054035 | FE_Hap29 | 0 | 0 | 0 | 2 | 0 | 2 |
| MN054036 | FE_Hap30 | 0 | 0 | 1 | 0 | 0 | 1 |
| MN054037 | FE_Hap31 | 0 | 0 | 21 | 0 | 0 | 21 |
| MN054038 | FE_Hap32 | 0 | 0 | 1 | 0 | 0 | 1 |
| MN054039 | FE_Hap33 | 0 | 0 | 1 | 0 | 0 | 1 |
| MN054040 | FE_Hap34 | 0 | 0 | 1 | 0 | 0 | 1 |
| MN054041 | FE_Hap35 | 0 | 0 | 2 | 0 | 0 | 2 |
| MN054042 | FE_Hap36 | 0 | 0 | 1 | 0 | 0 | 1 |
| MN054043 | FE_Hap37 | 0 | 0 | 1 | 0 | 0 | 1 |
| MN054044 | FE_Hap38 | 0 | 0 | 1 | 0 | 0 | 1 |
| MN054045 | FE_Hap39 | 0 | 0 | 0 | 0 | 7 | 7 |
| MN054046 | FE_Hap40 | 0 | 0 | 0 | 0 | 1 | 1 |
| MN054047 | FE_Hap41 | 0 | 0 | 0 | 0 | 1 | 1 |
| MN054048 | FE_Hap42 | 0 | 0 | 0 | 0 | 5 | 5 |
