# Supplementary material for: Relationships between crayfish population genetic diversity, species richness, and abundance within impounded and unimpounded streams in Alabama, USA
Source: PeerJ. 2024 Sep 24;12:e18006. doi: 10.7717/peerj.18006 (PMC11430169; doi:10.7717/peerj.18006)
Supplement: Supplemental Information 4 — These were used to estimate a genus-level phylogeny to identify outgroups when calculating Phylogenetic Diversity for F. validus and F. erichsonianus. [file peerj-12-18006-s004.docx]

**Supplemental Data S4.** The National Center for Biotechnology Information’s nucleotide database accession numbers for mtCOI from 71 representative species of *Faxonius*, and an outgroup (*Barbicambarus cornutus*). These were used to estimate a genus-level phylogeny to identify outgroups when calculating Phylogenetic Diversity for *F. validus* and *F. erichsonianus*.

| Accession Number | Genus and species | Accession Number | Genus and species |
| --- | --- | --- | --- |
| AY701215 | *Faxonius alabamensis* | KF773877 | *Faxonius mirus* |
| MG872956 | *Faxonius barrenensis* | KF773911 | *Faxonius mississippiens* |
| AF474346 | *Faxonius bisectus* | AY701223 | *Faxonius nais* |
| AF474347 | *Faxonius burri* | JX514455 | *Faxonius neglectus* |
| KU168750 | *Faxonius carolinensis* | KF773880 | *Faxonius obscurus* |
| KF773927 | *Faxonius chickasawae* | MG872945 | *Faxonius ozarkae* |
| KF773869 | *Faxonius cooperi* | AY701202 | *Faxonius pagei* |
| MG872936 | *Faxonius cristavarius* | MG872949 | *Faxonius palmeri* |
| AY701205 | *Faxonius deanae* | MG872951 | *Faxonius pardalotus* |
| AY701206 | *Faxonius difficilis* | KF773878 | *Faxonius perfectus* |
| MG872931 | *Faxonius durelli* | MG872938 | *Faxonius peruncus* |
| MN054045 | *Faxonius erichsonianus* | MG872953 | *Faxonius placidus* |
| AY701219 | *Faxonius etnieri* | ON792206 | *Faxonius propinquus* |
| MG872929 | *Faxonius eupunctus* | MG872948 | *Faxonius punctimanus* |
| MG872950 | *Faxonius forceps* | MG872935 | *Faxonius putnami* |
| AY701189 | *Faxonius harrisoni* | MG872941 | *Faxonius quadruncus* |
| AY701207 | *Faxonius hartfieldi* | AF474358 | *Faxonius rafinesquei* |
| AY701211 | *Faxonius hobbsi* | AY701224 | *Faxonius rhoadesi* |
| KF773923 | *Faxonius holti* | MG872921 | *Faxonius roberti* |
| MG872939 | *Faxonius hylas* | KT282432 | *Faxonius ronaldi* |
| AY701226 | *Faxonius illinoiensis* | KT282408 | *Faxonius rusticus* |
| JF438005 | *Faxonius immunis* | AF474360 | *Faxonius sanbornii* |
| KF773872 | *Faxonius indianensis* | AY701250 | *Faxonius saxatilis* |
| AF474351 | *Faxonius jeffersoni* | AF474367 | *Faxonius shoupi* |
| KF773906 | *Faxonius jonesi* | AY701197 | *Faxonius sloanii* |
| KT282428 | *Faxonius juvenilis* | AY701251 | *Faxonius spinosus* |
| KF773873 | *Faxonius kentuckiensis* | AF474361 | *Faxonius stannardi* |
| KF944434 | *Faxonius leptogonopodus* | AF474363 | *Faxonius tricuspis* |
| KT959445 | *Faxonius limosus* | MN054000 | *Faxonius validus* |
| AY701234 | *Faxonius longidigitus* | AF474364 | *Faxonius virginiensis* |
| JX514454 | *Faxonius luteus* | MG316159 | *Faxonius virilism* |
| KF827985 | *Faxonius macrus* | MG872926 | *Faxonius wagneri* |
| AY701208 | *Faxonius maletae* | KX238170 | *Faxonius williamsi* |
| MG872947 | *Faxonius marchandi* | AY701200 | *Faxonius wrighti* |
| AY701237 | *Faxonius medius* | MG872955 | *Faxonius yanahlindus* |
| KF773876 | *Faxonius meeki* | DQ113440 | *Barbicambarus cornutus* |
